# Relationships between types of UK national newspapers, illness classification, and stigmatising coverage of mental disorders

**DOI:** 10.1007/s00127-021-02027-7

**Published:** 2021-01-22

**Authors:** Yan Li, Rosanna Hildersley, Grace W. K. Ho, Laura Potts, Claire Henderson

**Affiliations:** 1 Florence Nightingale Faculty of Nursing, Midwifery and Palliative Care, King’s College London, London, UK; 2 Health Service and Population Research Department, Institute of Psychiatry, Psychology and Neuroscience, King’s College London, London, UK; 3 School of Nursing, The Hong Kong Polytechnic University, Hong Kong, People’s Republic of China; 4 Biostatistics and Health Informatics Department, Institute of Psychiatry, Psychology and Neuroscience, King’s College London, London, UK

**Keywords:** Stigma, Time to change, Newspapers, Diagnosis

## Abstract

**Background:**

Media coverage on mental health problems has been found to vary by newspaper type, and stigma disproportionately affects people with mental illness by diagnosis.

**Objective:**

This study investigated the relationships between types of UK national newspaper (tabloid vs. broadsheet), illness classification (SMI–severe mental illnesses vs. CMD–common mental disorders), and stigmatising coverage of mental disorders, and whether these relationships changed over the course of the Time to Change anti-stigma programmes in England and Wales.

**Methods:**

Secondary analysis of data from a study of UK newspaper coverage of mental illness was performed. Relevant articles from nine UK national newspapers in 2008–11, 2013, 2016 and 2019 were retrieved. A structured coding framework was used for content analysis. The odds an article was stigmatising in a tabloid compared to a broadsheet, and about SMI compared to CMD, were calculated. Coverage of CMD and SMI by newspaper type

was compared using the content elements categorised as stigmatising or anti-stigmatising.

**Results:**

2719 articles were included for analysis. Articles in tabloids had 1.32 times higher odds of being stigmatising than articles in broadsheet newspapers (OR 1.32, 95% CI 1.12–1.55). Odds of stigmatising coverage was 1.72 times higher for articles on SMI than CMD (OR  1.72, 95% CI 1.39–2.13). Different patterns in reporting were observed when results were stratified by years for all analyses. A few significant associations were observed for the portrays of stigmatising elements between tabloid and broadsheet newspapers regarding SMI or CMD.

**Conclusions:**

Tailored interventions are needed for editors and journalists of different newspaper types, to include specific strategies for different diagnoses.

## Introduction

Stigma is recognised as an important public health issue and a challenge for people with mental disorders globally [1]. Stigma has been interpreted as problems of knowledge (ignorance and misinformation), attitudes (prejudice) and behaviours (discriminations) [2]. Ample research shows that stigma negatively impacts the health and wellness of people with mental illness (e.g., lower access to healthcare, life expectancy, and self-esteem; increased social isolation and mood problems) [3, 4], and can incur various forms of social disadvantage (e.g., reduced opportunities for education, employment, and housing) [5, 6].

Stigma disproportionately affects people with different forms of mental illness [7]. Two main types of mental disorders, Severe Mental Illnesses (SMIs) and Common Mental Disorders (CMDs), are often studied [8]. SMIs include schizophrenia, psychotic disorders, and bipolar disorder; CMDs include depression, generalised anxiety disorder, panic disorder, obsessive–compulsive disorder, post-traumatic stress disorders. Research indicates that people with SMIs can suffer from higher levels of stigmatisation and discrimination compared with CMDs [9]. Specifically, many studies showed that people with schizophrenia are perceived by the public as being the most dangerous, violent and unpredictable compared to people with other mental disorder [10–12]. Because of this anti-stigma campaigns often target schizophrenia [13, 14]. On the other hand, stigmatisation is also experienced by individuals with CMDs [15], but generally to a lesser degree compared with illnesses with psychotic symptoms [16]. For example, one study showed that anxiety was seen most favourably by the public in comparison to other mental disorders as it was associated with less negative stereotypes and seen as more likely to be curable [17].

The media can influence public attitudes and play a significant role in raising awareness among citizens, empowering communities to take action, informing policymakers about pertinent social issues, and advocating for policy initiatives [18]. In particular, newspapers are a medium that is frequently and widely accessible and can reach a large number of people [19]. Therefore, newspapers are an important resource to educate the public and capture the attention of policymakers on a variety of issues related to mental illness, such as its treatment and intervention, as well as its costs and impacts on society (e.g., violence, suicide) [20]. However, biased, sensationalized, or inaccurate newspaper coverage on mental illnesses can adversely impact the public’s perception of people with mental disorders at large, and those with a SMI in particular. For example, prior research indicates that participants who recalled negative media coverage of mental illness were less likely to have the motivation to work with/live near people with mental disorders, and are more likely to believe that people with a mental illness are dangerous [21]. One study also showed that newspaper articles reporting crimes committed by people with schizophrenia are significantly longer and contained more stigmatising language than those describing other mental disorders [17].

The UK newspaper market is segmented into tabloid (also called ‘popular’) and broadsheet (also called ‘serious’), each serving a different readership [22]. UK tabloid newspapers (e.g. the Sun and the Daily Mirror) usually feature human interest stories, entertainment, sports, and scandal that are of interest to the general public, whilst broadsheet newspapers (e.g. Times, Guardian) often cater to readers in higher socioeconomic groups and engage them in debates about more serious public affairs (e.g., global economic, social, political issues) [23]. For the coverage on mental illness, tabloid newspapers might report ‘harrowing stories’ with often ‘lurid accounts’ of health consequences to increase the emotional impact of the news [24], whilst broadsheet newspapers tend to focus more on the official report and policies of health promotion [25]. Since different types of newspapers attract readers from different sectors of society, it is possible that differences in how mental illnesses are covered across these outlets can contribute to differences in levels of stigma in different demographic groups. Therefore, the relationship between types of newspaper (tabloid or broadsheet) and stigmatising coverage warrant empirical investigation.

The media have been a target of some long-running national anti-stigma programmes, including “Open Minds” in Canada [26], and Time to Change (TTC) in England [27]. The TTC programme is a continuing public anti-stigma campaign which aims to ‘inspire people to work together to end the discrimination surrounding mental health’ was launched in Jan 2009 in England, with its primary goal to reduce stigma through social contact and public education [27]. Time to Change has included protesting against incidents of particularly stigmatising coverage and work with journalists and editors comprising workshops on responsible coverage, and collaboration on the development of characters with mental illness portrayed in TV drama series [28]. The TTC anti-stigma programme provided general media guidelines to promote responsible reporting of mental illness in UK newspapers [27], and a media advisory service which includes general script advice for storylines featuring mental health problems and their own ‘mind your language’ section for journalists. From the evaluation of changes in UK newspaper coverage of mental illness from 2008 to 2016 in, there was a significant increase in the proportion of anti-stigma articles and a significant decrease in stigmatising articles over the years [28]. The overall coding of articles in 2016 revealed that reports on all diagnoses, except for schizophrenia, were more often anti-stigmatising than stigmatising [28]. However, it remains unclear whether and how this anti-stigma programme may have influenced reporting on different classifications of mental disorders (i.e. SMI vs CMD) over time or in different types of newspapers (i.e. tabloid vs broadsheet). Since the TTC interventions for journalists and editors were designed to promote more similar reporting between different types of newspapers, it is also useful to examine the changes in the relationships between stigmatising coverage and types of newspapers (i.e. tabloid or broadsheet) before and over the course of the TTC programme.

The present study aimed to examine the relationships between types of UK newspaper, disease classification, and stigmatising coverage regarding mental health problems over an 11-year period since the start of Time to Change. We hypothesized that (1) tabloids are more likely to have stigmatising coverage compared with broadsheets and the relationship will weaken over time; (2) newspaper articles featuring SMIs are more likely to have stigmatising coverage compared with CMDs and the relationship will weaken over time. We also conducted an exploratory analysis of the article content elements categorised as stigmatising or anti-stigmatising to compare tabloid and broadsheet coverage of both CMDs and SMIs.

## Methods

### Data source

We adopted a secondary analysis of the newspaper articles that were part of the evaluation of Time to Change. The Lexis Nexis Professional UK electronic newspaper database (www.lexisnexis.co.uk) was used to search through all articles from 18 local and national newspapers which were published in each study year (from 2008 to 2011, 2013, 2016, and 2019) on two randomly chosen days (including Saturday and Sunday) of every month, and which referred to mental illness.

In this study, we have analysed nine UK national newspapers including four broadsheet newspapers (Daily/Sunday Telegraph, Times/Sunday Times, Guardian/Observer, and Independent/Independent on Sunday), and five tabloid newspapers (Daily/Sunday Mail, Daily/Sunday Star, Daily/Sunday Express, Daily/Sunday Mirror, Sun/News of the World). This typology of UK national newspapers has been used in similar research to examine the newspaper readership in various demographic characteristics [25]. In this study, we adopted definitions of SMI and CMD from the UK Mental Health Foundation 2016 [29]. SMIs include schizophrenia and bipolar disorder, while CMDs include anxiety disorder, depression, post-traumatic stress disorder (PTSD), obsessive–compulsive disorder (OCD), agoraphobia, and postnatal depression.

### Search terms

Search terms consisted of 35 general and diagnostic terms covering the full range of mental disorders. The full texts of articles in the selected newspapers were searched using the following terms (* = wildcard): ‘mental health OR mental illness OR mentally ill OR mental disorder OR mental patient OR mental problem OR (depression NOT W/1 economic OR great) OR depressed OR depressive OR schizo* OR psychosis OR psychotic OR eating disorder OR anorexi* OR bulimi* OR personality disorder OR dissociative disorder OR anxiety disorder OR anxiety attack OR panic disorder OR panic attack OR obsessive–compulsive disorder OR OCD OR post-traumatic stress OR PTSD OR social phobia OR agoraphobi* OR bipolar OR ADHD OR attention deficit OR psychiatry* OR mental hospital OR mental asylum OR mental home OR secure hospital*.

### Inclusion and exclusion criteria

Articles were included if they focused on mental illness, i.e. upon people with such a condition or upon the services they receive. Articles that used a search term in a context unrelated to mental health (e.g. ‘the government is schizophrenic about this issue’), described a non-clinical use (e.g. ‘I’m feeling a bit depressed about this’) or in which diagnostic or slang terms were used metaphorically (e.g. ‘he’s driving me nuts’) were excluded. Articles relating primarily to developmental disorders such as autism, neurodegenerative diseases (e.g. Alzheimer’s disease) or alcohol/substance misuse alone were also excluded as these were not the focus of the TTC programme. Articles discussing other diagnoses or that did not mention a diagnosis were excluded from the analysis. If an article described both CMD and SMI, it was dropped from the analysis.

### Coding and the identification of elements

Newspaper articles were coded for their date, newspaper origin and article type (news, features, or opinion), as well as for any diagnoses mentioned. Articles published in different years were coded by different research assistants trained in the same way to use the same codebook. Each researcher coded a specific sample of articles published in 2008 using the coding framework, and their results were compared with those found by previous coders. Researcher agreement was quantified using a kappa agreement test [28], with the threshold set as 0.7, indicating substantial agreement. When the trainee researcher met this threshold, they continued to code the articles for inclusion in the sample. Any areas of uncertainty were discussed with CH and previous researcher until a consensus was reached.

The central theme conveyed in each article was coded into an ‘element’ which was ‘stigmatising, anti-stigmatising or neutral’. The elements were developed from existing studies or mental health reporting, the wider literature on mental health stigma and a process of inductive coding [28]. The coding aimed to identify the overall central message conveyed in each article and finally each article was classified as stigmatising, anti-stigmatising, mixed or neutral. Stigmatising themes include: danger to others, hopeless victim, strange behaviour, and the cause of their mental illness was their personal responsibility (e.g. due to the individual’s poor choices), sceptical of seriousness, or pejorative or inappropriate language. Anti-stigmatising themes include sympathetic portrayal (general public or public figure), causes of mental illness (genetic, psychosocial or other), recovery from or successful treatment of mental illness (pharmaceutical, psychosocial or other/not specified), mental health promotion, stigma, injustice and prevalence. These are separate codes that are included in the dataset, whereas the distinction between general public/public figure, genetic/psychosocial causes and pharmaceutical/psychosocial treatments were subsets of the sympathetic portrayal, causes of MI and treatment of MI codes. Articles in which both stigmatising and anti-stigmatising elements were given equal weight (for example, both as primary elements) were coded as “mixed”. When no element was present, the article was coded as “neutral”.

A detailed codebook was established to guide the coding process. Finally, each article was coded overall as stigmatising, anti-stigmatising, mixed or neutral based on the overall weight (where the elements appeared and in how much of the article they appeared) and key/central messages conveyed by the article. As we adopted a secondary analysis of the coded newspaper articles from 2008 to 2019, more details of the coding process could be seen in the team’s previous publications [28, 30], and request to CH. Only articles relating to national newspapers that discussed either SMI or CMD (not both) were included in the analysis for this study.

### Statistical analysis

Data were entered and analysed in Stata version 16.0. Frequencies and proportions (*n*, %) were used to describe overall elements (stigmatising, anti-stigmatising, mixed or neutral), newspaper type (tabloid or broadsheet) and illness diagnosis featured in the articles by year. The first hypothesis was tested by calculating the odds an article was stigmatising in a tabloid paper compared to a broadsheet. The second hypothesis was tested by calculating the odds an article was stigmatising in SMI compared to CMD. The hypotheses on the changes of the relationships over years were assessed by stratifying both analyses by year and pooling the overall odds ratio using the Mantel–Haenszel method. The exploratory analysis on the sample of articles to identify associations between the presence of an article content element and the newspaper type (tabloid vs broadsheet) was carried out using chi-squared tests separately for CMDs and SMIs. Fisher’s exact tests were used when cell counts were less than 5.

## Results

### The sample

**Fig. 1:**
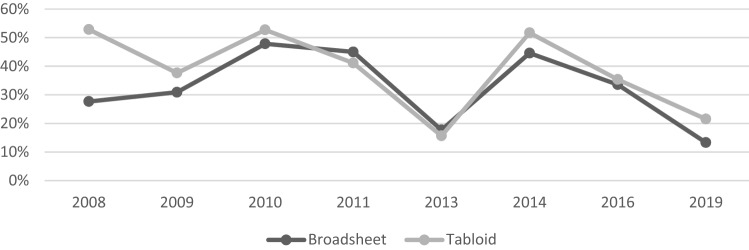
Proportion of articles that were stigmatising in tabloid and broadsheet papers, by year from 2008 to 2019

**Fig. 2:**
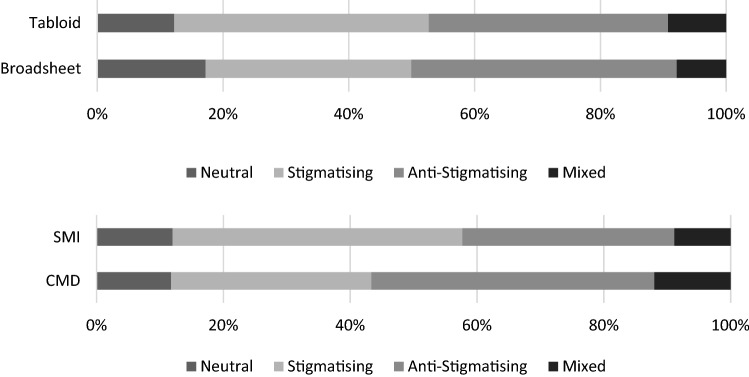
Proportion of articles that are stigmatising, anti-stigmatising, and neutral or mixed by diagnosis and newspaper type from 2008 to 2019

A total of 2719 articles were included in the analysis, with 1320 (48.5%) articles from broadsheet newspapers, and 1399 (51.5%) from tabloids collected in the years 2008, 2009, 2010, 2011, 2013, 2014, 2016 and 2019 (Table 1). Overall, 34.6% of articles were coded as stigmatising, with 31% of broadsheet articles and 38% of tabloid articles being coded as stigmatising, although these proportions varied in the period from 2008 to 2019. Overall, 43.8% of articles in the sample were coded as anti-stigmatising, ranging from 35 to 67% anti-stigmatising over the period. Articles regarding SMIs (schizophrenia or bipolar) only made up 16.5% of the sample. The number of newspaper articles featuring CMD (*n* = 2270, 83.5%) is nearly five times the number of articles featuring SMI (*n* = 449, 16.5%). Figure 1 shows the proportion of stigmatising articles in the sample over the years. There is an overall decreasing pattern for the stigmatising coverage for both broadsheet and tabloid newspapers, with a marked drop in stigmatising coverage in 2013 (Fig. 1), which rose again in 2014, then steadily fell in 2016 and 2019. The proportions of articles that were stigmatising, anti-stigmatising, mixed or neutral, separated by CMD and SMI, as well as tabloid and broadsheet newspapers, are shown in Fig. 2.

**Table 1:** Frequency and the proportion of newspaper type and diagnostic category represented in the sample, by year

Year	2008		2009		2010		2011		2013		2014		2016		2019		Total 2719	
Total	315		311		286		261		429		371		400		346			
	*n*	%	*n*	%	*n*	%	*n*	%	*n*	%	*n*	%	*n*	%	*n*	%	*n*	%
Overall category																		
Neutral	32	10.2	28	9.0	24	8.4	28	10.7	97	22.6	34	9.2	42	10.5	44	12.7	329	12.1
Stigmatising	131	41.6	106	34.1	144	50.3	112	42.9	72	16.8	178	47.9	139	34.7	59	17.0	941	34.6
Anti-Stigmatising	129	40.9	144	46.3	100	35.0	108	41.4	149	34.7	132	35.6	198	49.5	231	66.8	1191	43.8
Mixed	23	7.3	33	10.6	18	6.3	13	5.0	111	25.9	27	7.3	21	5.3	12	3.5	258	9.5
Newspaper type																		
Broadsheet	141	44.8	165	53.1	140	49.0	120	46.0	231	53.8	195	52.6	140	35.0	188	54.3	1320	48.5
Tabloid	174	55.2	146	46.9	146	51.0	141	54.0	198	46.2	176	47.4	260	65.0	158	45.7	1399	51.5
Diagnostic category																		
Severe mental illness*	53	16.8	66	21.2	47	16.4	57	21.8	77	17.9	49	13.2	76	19.0	24	6.9	449	16.5
Common mental disorder**	262	83.2	245	78.8	239	83.6	204	78.2	352	82.1	322	86.8	324	81.0	322	93.1	2270	83.5

*SMIs include schizophrenia and bipolar disorder, **CMDs include anxiety disorder, depression, Traumatic Stress Disorder (PTSD), Obsessive–Compulsive Disorder (OCD), Agoraphobia, postnatal depression

### Relationships between types of newspaper and stigmatising coverage

As shown in Table 2, articles in tabloid newspapers have 1.32 times higher odds of being stigmatising compared with articles in broadsheet newspapers, after adjusting for year of publication (95% CI 1.12–1.55). While the adjusted odds ratio was statistically significant overall between the two types of newspaper, a statistically significant different pattern in reporting was only observed in 2008 and 2019 when results were stratified by year.

**Table 2:** Odds ratio that an article is stigmatising, stratified by year using the Mantel–Haenszel method

Year	Odds ratio	[95% confidence interval]	
		Upper limit	Lower limit
Odds that a stigmatising article is in a tabloid vs a broadsheet (baseline), by year published			
2008	2.93*	1.80	4.79
2009	1.35	0.84	2.17
2010	1.22	0.76	1.94
2011	0.85	0.52	1.40
2013	0.86	0.52	1.43
2014	1.33	0.88	2.00
2016	1.08	0.70	1.67
2019	1.79*	1.01	3.16
Overall OR Adjusted for year	1.32*	1.12	1.55
Odds that a stigmatising article is about SMI vs CMD (baseline), by year published			
2008	2.08*	1.14	3.80
2009	2.17	1.24	3.82
2010	1.41	0.75	2.65
2011	1.15	0.64	2.08
2013	1.13	0.59	2.15
2014	1.39	0.76	2.55
2016	2.06*	1.24	3.45
2019	5.85*	2.42	14.17
Overall OR Adjusted for year	1.72*	1.39	2.13

*CMD* common mental disorders (including anxiety disorder, depression, PTSD, OCD, Agoraphobia, postnatal depression); *SMI * severe mental illness (including bipolar/manic depression, schizophrenia)

*Statistically significant

### Relationships between different diagnostic groups and stigmatising coverage

In terms of the illness diagnosis, articles featuring SMI have 1.72 times higher odds of being stigmatising compared with articles featuring CMD after adjusting for year of publication (95% CI 1.39–2.13, detailed presented in Table 2). While the adjusted odds ratio was statistically significant overall between SMI and CMD, a statistically significant different pattern in reporting was only observed in 2008, 2009, 2016 and 2019 when results were stratified by year.

### Coverage of CMD and SMI by newspaper type

Articles (details presented in Table 3) in tabloid newspapers reported significantly more “danger to others” than those in broadsheet newspapers in both SMI and CMD. For articles about CMD, tabloid newspapers contained fewer antisigmatising element (i.e., prevalence of mental illness) than broadsheet newspapers. There are no significant differences about other elements between these two types of newspapers.

**Table 3:** Elements in tabloid versus broadsheet newspaper articles covering CMD or SMI

	CMD (*N* = 2270)			SMI (*N* = 449)		
	Articles in broadsheets *N* = 1083	Articles in tabloids, *N* = 1187	Chi squared, *p* value	Articles in broadsheets *N* = 237	Articles in tabloids, *N* = 212	Chi squared (*p* value)
	*N* (%)	*N* (%)		*N* (%)	*N* (%)	
Stigmatising elements						
Danger to others	76 (6.8)	138 (11.6)	13.31, *p* < 0.01*	36 (15.2)	48 (22.6)	4.09, *p* = 0.04*
Problem for others	69 (6.2)	84 (7.1)	1.32, *p* = 0.25	19 (8.0)	25 (11.8)	1.80, *p* = 0.18
Hopeless victim	159 (14.2)	202 (17.0)	3.441. *p* = 0.06	42 (17.7)	32 (15.1)	0.17, *p* = 0.68
Strange behavior	115 (10.3)	138 (11.6)	2.14, *p* = 0.14	45 (19.0)	35 (16.5)	0.47, *p* = 0.49
Personal responsibility for the cause	55 (4.9)	80 (6.7)	2.50, *p* = 0.11	15 (6.3)	12 (5.7)	0.09, *p* = 0.77
Sceptical of seriousness	33 (2.9)	40 (3.4)	0.12, *p* = 0.72	4 (1.7)	4 (1.9)	1, *p* = 0.76^a^
Pejorative language	33 (2.9)	55 (4.6)	3.41, *p* = 0.06	26 (10.9)	32 (15.1)	1.69, *p* = 0.19
Anti-stigmatising elements						
Sympathetic portrayal of people with mental illness	312 (27.9)	327 (27.5)	0.38, *p* = 0.54	39 (16.5)	46 (14.6)	2.00, *p* = 0.16
Causes of mental illness (genetic, psychosocial etc.)	256 (22.9)	263 (22.2)	0.62, *p* = 0.43	24 (10.1)	31 (14.6)	2.10, *p* = 0.15
Recovery or treatment of mental illness	185 (16.5)	160 (13.5)	2.05, *p* = 0.15	16 (6.8)	19 (8.9)	0.76, *p* = 0.38
Mental health promotion	86 (7.7)	98 (8.3)	0.26, *p* = 0.61	15 (6.3)	18 (8.5)	0.77, *p* = 0.38
Mental health stigma	33 (2.9)	38 (3.2)	0.01, *p* = 0.93	7 (2.9)	4 (1.9)	0.56, *p* = 0.85^a^
Injustice faced by people with mental illness	73 (6.5)	66 (5.6)	1.17, *p* = 0.28	23 (9.7)	19 (8.9)	0.07, *p* = 0.79
Prevalence of mental illness	65 (5.8)	47 (4.0)	4.61, *p* = 0.03*	10 (4.2)	5 (2.4)	1.20, *p* = 0.27

One newspaper might contain more than one element

*CMD*  common mental disorder; *SMI* severe mental illness

^a^Fisher ‘’s exact test

*Significant at the *p* < 0.05 level

## Discussion

To our knowledge, our study is the first to investigate the relationships between stigmatising coverage, newspaper type (tabloids vs broadsheet), and mental disorder classification (SMI vs CMD) in the UK. Articles analysed in the present study are from UK national newspapers that were published immediately before and over the course of three phases of the TTC anti-stigma programme (from 2008 to 2019). Our study findings support the research hypotheses that tabloid newspapers are significantly more likely to have stigmatising coverage than broadsheet newspapers, and articles featuring SMI are more stigmatising than those featuring CMD, although the effect is not consistently observed in each year studied. Our exploratory analyses suggest that this is not because of a relatively greater focus on SMI in tabloids; rather some aspects of their coverage of CMD and SMI are more likely to be stigmatising.

Our study findings are consistent with previous research in the analysis of Canadian [31] and UK newspapers [32] that articles published in broadsheet newspaper were more positive than those published in tabloid newspapers. Prior research indicated that tabloid journalists tend to report mainly on early signs and symptoms of illnesses, whereas broadsheet newspapers provided more comprehensive information covering illness characteristics and health promotion [33]. Also, tabloid newspapers tend to report harrowing stories to increase the emotional impact of the news and attract readers as well as increase sales [24]. These differences increase the chances of stigmatising language in tabloid newspapers. As tabloid newspapers’ audience tend to have lower socioeconomic status stigmatising reporting in tabloid newspapers may contribute to the socioeconomic differential found in stigma-related knowledge, attitudes and desire for social distance found in general population surveys in England [34, 35]. The findings of this study imply that Time to Change has had positive effects in reducing stigma as shown in previous newspaper analyses as well as findings from a general population survey [29, 36] and a survey of people using mental health services [37]. Though stigmatising coverage in all types of newspapers is decreasing, the differences between tabloid and broadsheet newspapers remained statistically significant in 2019.

The pattern of stigmatising coverage in different diagnostic groups is consistent with previous research on public perceptions of various mental illnesses were often judged by the public as more ‘serious’ and often linked with more negative stereotypes and viewed as more dangerous and less likely to recover in comparison to CMDs [13]. Psychotic symptoms are linked with ‘mad’ and violent behaviour by the public, although it is evident that most persons with schizophrenia are not violent or ‘dangerous’ [38]. Research evidence also shows that people diagnosed with schizophrenia represent lower levels of violence when compared to those diagnosed with affective disorders, substance use disorders or personality disorders [38]. Depressed people are often linked with “lazy and difficult to communicate with” [13, 39]. The news media might reinforce the stigma by focusing on reporting those negative consequences reported [40].

## Limitations

Several limitations need to be considered when interpreting the findings. Our study only includes newspapers, the other media including magazines, broadcast media and social media (e.g., Facebook, YouTube), as well as online news, might also contain rich information that drives public perceptions of mental illness and warrant further exploration. Also, newspapers might not be the most accurate measure of media influence on public attitudes as newspaper consumption has decreased over the period of Time to Change. Content analysis was used only for the text and therefore other articles’ information (e.g., photographs, pictures places of articles/headlines) might be missed. The newspapers were analysed by different trained research assistants for each year, though the same detailed codebook was used and agreement between coders was good [28].

## Conclusions and implications

Newspapers are a powerful tool in shaping the public’s opinion and attitude towards mental disorders, disseminating knowledge and educating the general public, as well as informing policymakers on mental health issues and help-seeking behaviours [41]. Efforts are needed to strengthen the quality and accuracy of the presentation of mental health issues in the media. Previous anti-stigma programmes (e.g., ‘Opening Minds’ in Canada and the ‘Time to Change’ programme in England) established media reporting guidelines or advisory services, however, those reporting guidelines were general advice without providing tailored guidelines for different types of media or diagnosis.

Given the wide range of stigmatising coverage in news media as well as various levels of stigmatisation reported in different types of newspaper, tailored anti-stigma interventions and guidelines need to be designed for different types of newspapers to establish more responsible and accurate reporting guidelines for mental health problems in the future. As broadsheet newspapers are often read by people who are more likely to work in the government context and have the power to change certain policy guidelines, efforts can be made to incentivise broadsheet newspaper journalists to cover more comprehensive reporting of mental illness, with the description of the events/illness case, and together with ways to improve the public’s acceptance and lessen the biased view for people with mental health problems. Since tabloid newspapers had the most negatively oriented coverage of mental illness, specific and more intensive training and interventions might be needed for the journalists and editors of tabloid newspapers, and they are also encouraged to gain ideas and real experiences from people with mental disorders.

As Tabloid newspapers (e.g., the Sun and the Daily Mail) have larger circulation than broadsheet newspapers (e.g., the Times and the Guardian) [42], those training for journalists and editors of tabloid newspapers could reach wider groups of people to reduce the stigmatising reporting of mental illness as well as disseminate mental health knowledge. We also recommend that the contact-based education provided by TTC during its second phase for journalists and editors of newspapers could be continuously implemented to allow for staff turnover and reversion to previous styles of coverage. Also, the explanations of the aetiology and symptoms of mental illness, as well as the reasons why the stigma/prejudice to this group of people might impact negatively to the treatment/health consequences of this group are also needed for both tabloid and broadsheet newspaper staff. It is, therefore, necessary for anti-stigma interventions to address different misconceptions and levels of stigma towards people with SMI and CMD. Some common messages used in anti-stigma campaigns such as “mental illness might affect everyone, and they are treatable’ might not be sufficient for all diagnoses [40].

Future research is suggested to examine all possible contributing factors to the difference of stigmatising reporting between tabloid and broadsheet newspapers, as well as SMI and CMD diagnostic groups. The contributing factors can provide valuable guidance for the tailored interventions proposed above. Besides, as more and more people get news from social media than newspapers in recent years [43], further analysis of stigmatising and anti-stigmatising reporting are also suggested to target on e-resources including online news and some popular social media platforms (e.g., Facebook and YouTube).

## Data Availability

All data will be shared on reasonable request made to Dr Henderson.
